# Understanding Internal Accountability in Nigeria’s Routine Immunization System: Perspectives From Government Officials at the National, State, and Local Levels

**DOI:** 10.15171/ijhpm.2016.150

**Published:** 2016-12-10

**Authors:** Daniel J. Erchick, Asha S. George, Chukwunonso Umeh, Chizoba Wonodi

**Affiliations:** ^1^Department of International Health, Johns Hopkins Bloomberg School of Public Health, Baltimore, MD, USA.; ^2^School of Public Health, University of the Western Cape, Cape Town, South Africa.; ^3^i+Consortium, Abuja, Nigeria.; ^4^International Vaccine Access Center, Johns Hopkins School of Public Health, Baltimore, MD, USA.

**Keywords:** Vaccines, Immunization, Health Systems, Accountability, Nigeria

## Abstract

**Background:** Routine immunization coverage in Nigeria has remained low, and studies have identified a lack of accountability as a barrier to high performance in the immunization system. Accountability lies at the heart of various health systems strengthening efforts recently launched in Nigeria, including those related to immunization. Our aim was to understand the views of health officials on the accountability challenges hindering immunization service delivery at various levels of government.

**Methods:** A semi-structured questionnaire was used to interview immunization and primary healthcare (PHC) officials from national, state, local, and health facility levels in Niger State in north central Nigeria. Individuals were selected to represent a range of roles and responsibilities in the immunization system. The questionnaire explored concepts related to internal accountability using a framework that organizes accountability into three axes based upon how they drive change in the health system.

**Results:** Respondents highlighted accountability challenges across multiple components of the immunization system, including vaccine availability, financing, logistics, human resources, and data management. A major focus was the lack of clear roles and responsibilities both within institutions and between levels of government. Delays in funding, especially at lower levels of government, disrupted service delivery. Supervision occurred less frequently than necessary, and the limited decision space of managers prevented problems from being resolved. Motivation was affected by the inability of officials to fulfill their responsibilities. Officials posited numerous suggestions to improve accountability, including clarifying roles and responsibilities, ensuring timely release of funding, and formalizing processes for supervision, problem solving, and data reporting.

**Conclusion:** Weak accountability presents a significant barrier to performance of the routine immunization system and high immunization coverage in Nigeria. As one stakeholder in ensuring the performance of health systems, routine immunization officials reveal critical areas that need to be prioritized if emerging interventions to improve accountability in routine immunization are to have an effect.

## Background

Interest in accountability in health is rapidly rising, with several reviews recently published, ^1-5^ multiple donor and civil society networks organized,^6,7^ research initiatives underway,^8^ and a global strategy for women’s, children’s, and adolescent’s health, with a strong focus on accountability, launched in 2010 by the United Nations Secretary-General.^9,10^ Most efforts to address accountability in health systems have focused on external accountability, ie, how communities interact with the system to improve responsiveness.^7,11,12^ However, internal accountability, which involves the interactions of technical, managerial, and political actors within government, is of equal importance, and plays a foundational role in the quality of healthcare services.^3,13,14^

In Nigeria, accountability is also a concern for health services, including immunization.^12,15^ Routine immunization coverage in Nigeria has fluctuated in recent years, with only 38% of children reached with three doses of the DPT3/Penta3 vaccine (Nigeria Demographic and Health Survey [DHS] 2013); this compares to 68% in neighboring Cameroon (DHS 2011), and 89% in nearby, high-performing Ghana (DHS 2014).^16-18^ There is significant heterogeneity in the strength of routine immunization systems among Nigerian states, and studies have attributed poor performance to accountability challenges crosscutting governance, service delivery, finance, human resources, logistics, and data management.^19-22^

Nigeria has a decentralized healthcare system, with responsibility for tertiary, secondary, and primary healthcare (PHC) assigned to the federal, state, and local levels, respectively. Management of the delivery of primary healthcare services, including immunization, falls to local governments, which typically have the least resources and capacity.^23^ State governments provide oversight and support in PHC delivery, for example, through vaccine stock management and health facility supervision. Federal government agencies, including the Federal Ministry of Health and National Primary Healthcare Development Agency (NPHCDA), are tasked with providing technical assistance and policy direction.

In an attempt to eliminate accountability challenges associated with the fragmentation of service delivery across levels of government, in 2011, Nigeria rolled out the Primary Health Care Under One Roof initiative in line with the National Council on Health’s 2010 recommendation.^24^ This initiative aims to improve ownership and lines of authority by integrating all aspects of primary healthcare – finance, management, and implementation – under one state level authority, the State Primary Healthcare Development Agency.^25^ Several Nigerian states have already established such institutions, although the status of their implementation, and the extent to which they meet the guidelines set forth by NPHCDA, varies greatly.^26^

In 2013, health officials and civil society partners in Nigeria developed the Accountability Framework for Routine Immunization (AFRIN) in Nigeria, which defines roles, responsibilities, and reporting structures for the routine immunization system.^27,28^ AFRIN also outlines rewards and sanctions to help enforce these responsibilities, and indicators for monitoring the performance of the system. The development of AFRIN is a critical step towards improving accountability in the routine immunization system. However, AFRIN still needs to be operationalized, and strategies to implement AFRIN are under development.

To aid AFRIN’s future implementation, we undertook this qualitative study to understand the views of immunization and health officials on the challenges to internal accountability and barriers to high performance in the immunization system across the national, state, local, and health facility levels in Nigeria.

## Methods

This study was conducted primarily in Niger State in the north central geopolitical zone of Nigeria, with additional interviews of national officials occurring in Abuja, Federal Capital Territory. Niger State is mostly rural and agrarian with a population of roughly 4 million people living in 25 local government areas (LGAs).^29^ Delivery of immunization is the responsibility of the State Ministry of Health and State Primary Healthcare Development Agency, and occurs at nearly all of the state’s 1322 public and privately owned PHC centers, 12 secondary facilities, and a single tertiary facility, although the majority of the burden falls to PHC centers, which are often understaffed and underequipped.^30^ Immunization coverage in Niger State is low and varies across LGAs. Among children aged 12-23 months, three dose DPT3/Penta3 coverage in Niger State was 37% in 2013.^16^

Following an exploratory aim, our study qualitatively interviewed 17 government health officials and healthcare workers involved in routine immunization activities at the national, state, LGA, and health facility levels (Table). Purposive sampling was utilized to capture maximum variation in experience across the routine immunization system and within each level of government.

**Table:** Demographic and Employment Characteristics of Study Participants

	** National**	** State**	** LGA**	** HF**
Respondents	4	3	4	6
Age^a^	46	41	45	37
Proportion male	100%	100%	75%	33%
Years worked in healthcare^a^	21	12	21	9
Years worked in immunization^a^	12	10	12	8
Years worked in current position^a^	3	5	6	3

Abbreviations: LGA, local government area; HF, health facility. ^a^ Mean.

Interviews were framed as a discussion of issues related to accountability and routine immunization for the purpose of developing strategies to improve performance of the Nigerian system. Interview guides were structured according to an accountability framework previously developed by our research team, which was informed by a literature review on how and why accountability initiatives work.^31^ This framework organizes elements of accountability into three axes based upon how they drive change in health systems. The *axis of ability* supports change by enabling service delivery actors with formal rules outlining roles, responsibilities, and standards that appropriately expand their authority to act, and the informal norms and inputs that support change in performance. The *axis of power* sparks change by wielding ‘sticks’ that curb their potential abuse of power or neglect of duty, and also by offering ‘carrots’ that incentivize the constructive agency of service delivery actors. Lastly, the *axis of justice* steers the strategic direction of change by balancing political leadership, community ownership, and social equity, so that accountability efforts support progressive change, rather than being captured by self-interests ( Figure ).^31^

**Figure:**
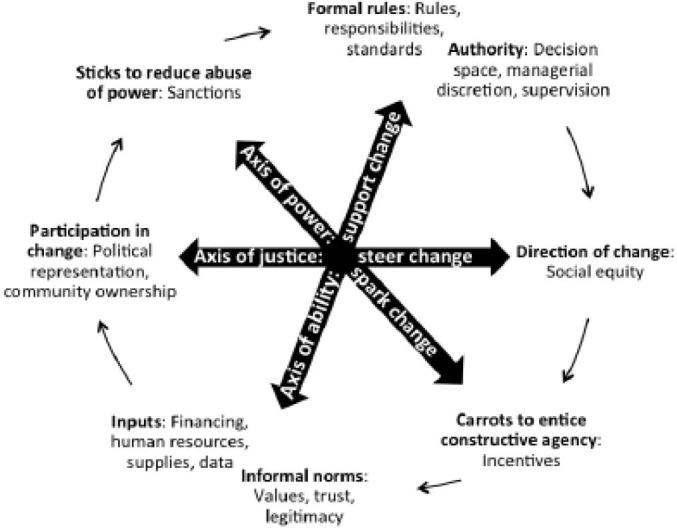
Conceptual Framework: Dynamic Dimensions of Accountability in Health Systems Along the Axes of Power, Ability, and Justice (from George et al^31^).

Semi-structured individual interviews, lasting 30-60 minutes, were conducted in English and digitally recorded. Interviews were transcribed, entered into NVivo™ 10(QSR International) and reviewed using thematic content analysis. Responses were coded following an initial codebook, based upon our nine accountability domains and sub-concepts within those domains. After testing the coding structure on a few key transcripts, the codebook was revised and applied to both subsequent and previously coded segments.^32,33^ Common themes emerging from the data were mapped according to each area of accountability in our framework.

## Results

Results are presented according to elements of accountability in Figure, including the axis of ability: formal rules, informal norms, authority, and inputs; the axis of power: carrots to entice constructive agency and sticks to reduce abuse of power or neglect of duty; and the axis of justice: political leadership, community ownership, and social equity. Quotes in the text are referenced by an anonymous identifier, indicating the level of government and respondent number (H = health facility, L = LGA, S = state, and N = national).

### Axis of Ability

#### Formal Rules: Roles, Responsibilities, and Standards

Many of the health and immunization officials surveyed had job responsibilities focused exclusively on routine immunization, particularly at higher levels of government (national and state). At lower levels, especially at the health facility level, respondents tended to have multiple responsibilities related to delivery of PHC, of which routine immunization and mass immunization campaigns were only a part.

At the national and state level, provision of an appointment letter, job description, or list of responsibilities occurred in some cases, in contrast to the LGA and health facility levels. Most officials at the LGA and health facility levels were given only verbal instructions about their role and responsibilities at the start of employment. Several respondents had already been working in the healthcare system when they were offered their current position, and moved from one position to the next, sometimes without sufficient orientation.

At the national and state levels, reported use of guidelines and standard operating procedures was more common than at LGA and health facility levels, where few had written guidelines or standard operating procedures for their work, although some participants reported using standing orders for clinical care. When asked if they were provided any guidelines for their work, one state official responded, “not specifically, most of the things I learn on the job and [in] training (S1).” Another state official reported using guidelines for the administration of immunizations, as well as guidelines for the management of mass immunization campaigns. Several respondents said that it would be helpful to have written guidelines for their responsibilities, especially in cases where their supervisor is unavailable or mistakenly provides incorrect information. One national level respondent explained, “health is a collective responsibility…but if you don’t put clear plans on ground to define roles…there will be confusion and wasted effort, and that has played [out] over the year while we were trying to establish guidelines and standards for people to follow (N3).”

When asked about what performance standards or targets exist in their work environment, health facility respondents typically referred to the number of immunization sessions held. At the LGA level, officials focused on submitting their monthly reports on time. Several officials mentioned immunization coverage targets as important benchmarks for performance.

A few respondents stated that the lack of formalized responsibilities leads to difficultly in holding staff accountable. One health facility official suggested that a schedule of duties would improve accountability, and a national level official stated that periodic reorientation on roles and responsibilities were needed for health workers in the immunization system. Despite the lack of formalized responsibilities, all respondents could explain in very general terms the basic responsibilities associated with their jobs. Many officials directly recommended clarifying roles and responsibilities as a possible intervention to improve accountability and performance in Niger State’s routine immunization system.

#### Informal Norms

Although interviews focused primarily on formal accountability areas, respondents did raise some issues related to informal norms in the context of the other elements of our accountability framework. At the national level, there was an explicit recognition of the presence of informal norms. Commenting on the start of their current position, one official stated, what “I actually met was series of traditions that [the] people that occupied the table before me had undertaken, [but] I try to define the way I see the job [to be] more impactful (N3).”

#### Authority: Decision Space and Managerial Discretion

Responses to questions regarding decision space and managerial discretion concerned the ability of supervisors to discipline poorly performing supervisees, and the inability of supervisors to access funding for activities. For example, a health facility and several LGA officials spoke about not having the authority to recommend the transfer of employees that do not perform to expected standards or refuse to cooperate.

Officials at all levels of the immunization system mentioned the need for more authority to access and disburse funding for routine immunization activities. Without this authority, they face delays in service delivery while waiting for funding, and often waste time because they have to visit their supervisors “cap-in-hand everyday” until funding is released (S1). Similarly, state officials reported not having sufficient authority to release vaccines and supplies, such as immunization data collection forms, which are used to track the number of vaccines delivered to each health facility. Another state official relayed that in the face of delays in provision of data collection forms from the national government, officials “always improvise,” and “instead of having all of the data tools available, they spend out of their own money” to photocopy forms (S3).

An LGA official reported that when there are problems, like vaccine shortages, they often reach out to local political leaders, such as the LGA chairman (the highest elected official at the LGA level). In some cases, political leaders will resolve the problem through their authority and network of personal influence. Other times, immunization officials will not receive any response from these leaders, and will be forced to wait until higher levels of government take action. One LGA official explained that sometimes if an action is taken without consulting the LGA chairman, the chairman will respond by saying that “you are becoming too powerful for him (L3).” In another instance, officials could not act without the chairman, who was out of town, and were delayed until they were able to reach him by text message, at which point the chairman helped to sort out their problem.

To alleviate delays created by waiting for the action of supervisors and political leaders, a health facility official suggested direct “allocation [of vaccines and supplies] to every facility…from the state (H3).” Similarly, at the state level, respondents suggested decentralizing decision-making and “delegating authority,” citing instances when they must “wait for an authority to approve transportation, logistics,” which “sometimes takes one, two, three days,” and negatively impacts their work (S3, S1). Another state official said, “there should be a structure where the request (for generator fuel) doesn’t have to come to me. [LGA officials] can be empowered to handle some responsibilities too (S2).” An LGA level official explained that they had previously acted without approval, only to be reprimanded later. When asked about ways to prevent this type of conflict over authority with a supervisor, the official offered, “try to be…transparent, respect must [be present], and we must…avoid a communication gap between us (L4).”

While most officials reported frustration with delays due to inability to authorize spending, one national level immunization official also noted that communication with the community could improve if there was “more freedom to…talk to the media about our programs [without having] to wait to get clearance from higher authority (N3).”

#### Supervision

Officials across levels of government reported an inability to conduct sufficient supervision of facilities and staff under their management. State officials commented on not having enough time, transportation, or funds to go out on supervision visits. In response to a question about what could be done to improve supervision, one official responded, “first and foremost is the logistics, in terms of transportation. Funds for [supervision visits]…[are] totally unavailable (S1).” Without these supervision visits, state officials have no way of knowing whether LGA officials are properly visiting (at least one visit per health facility per month) and supervising the health facilities under their jurisdiction. One LGA official said that with money for transportation they could visit officials from rural areas because when these officials “see new faces…[they] take it more seriously, and when [we] do [this supervision] regularly, it will improve the work (L1).”

Beyond the frequency of supervision, the purpose, content, and quality of supervision differed across levels of government. Several health facility officials stated that their interactions with their supervisors, typically officials from the LGA level, were limited solely to reporting when vaccines are low and requesting reimbursements for transportation. Other officials reported that when they are able to interact with their supervisor, for example, by reviewing routine immunization session plans or ledgers for storing vaccine stock balances, they learned from the interaction, and that it helped with problem solving. One LGA official explained that supervision “helps to solve problems, [and] the instruction [supervisors] give, I pass on to my colleagues in the field (L2).” A state official offered, “the feeling of knowing that someone is there to help solve a problem helps me a lot (S2).” Officials seemed to report problems to their supervisors or political officials as they arose, typically through informal, verbal means of communication, either in person or by phone or text message, when collecting vaccines from the store or during a supervision visit. However, officials would sometimes face a long wait until the problem is resolved or find it is never addressed.

Officials described supervision as able to generate motivation for high performance among supervisees and foster an environment of improved communication. Officials found it motivating when supervisors respond to their requests and consult with their supervisees in the decision-making process. An LGA official reported that supervision has the effect of “making me face my work with seriousness (L1).” Some officials reported that they reach out to their supervisors when their responsibilities were unclear. For example, a local official said, “it is for my superior to guide me on the job.… What I don’t know, I will ask my superior officer to tell me (L2).” Another LGA official pointed to the need to develop supervision guidelines for managers, saying that when “you are going out to supervise and you don’t know what you [need to] supervise, then [your effort is] rubbish (L4).” Negative behaviors of supervisors were reported by respondents to be demotivating, including when supervisors promise to visit a health facility but do not follow through, or when they get angry and shout at supervisees.

#### Inputs

Our interview guides explored a range of inputs, such as vaccines, supplies, funding, data, and human resources, all required to deliver immunization services.

##### 1. Funding

Respondents reported insufficient or late funding disbursements for PHC activities, including immunization, to be a major impediment to their ability to carryout their activities, and many listed specific examples of activities that were interrupted because of unavailable funding. Mobility was closely tied to funding, whether through absence of a vehicle, fuel, or cash for public transport, and was reported as a common impediment to vaccine collection, delivery, and supervision across government. LGA and health facility officials said they take steps to deliver vaccines and fulfill their responsibilities despite not having the required funding. Respondents emphasized that consistent availability of funding would lead to increased productivity.

At health facilities, activities affected by unavailability of funding included vaccine collection and outreach sessions. One health facility respondent explained that when there is no money for fuel, they use their “legs to get to some of the villages surrounding” their office (H3). In response to probing about how they reach farther villages, the respondent answered, “to be realistic, what you are supposed to do cannot be done at times, even though you try (H3).” At the LGA level, challenges included funding for transportation for health facility supervision and cold chain equipment maintenance. An LGA official explained, “presently one of the fridges is bad and here there is no money to repair it (L2).” When probed about whether they had reported this problem, the respondent said, “they have heard [about] it, and they have to think of a way forward,…unless money comes,” nothing will be done (L2). Another explained, “when there is no [power] and we can’t use the generator [because there is no funding] we sometimes drive to the state capital…to get ice packs, and in two or three days we come again for more (L1).” This reduced the time available for officials to spend on supervision.

##### 2. Human resources

At the LGA and health facility levels, too few staff was also a frequently reported problem. One health facility respondent seemed satisfied with the number of staff at their facility, but others reported that the number of staff in their facility was insufficient for their routine immunization activities and the number of people they typically serve. Insufficiencies in staff capacity and distribution contribute to this problem, for example, a health facility respondent explained that although they have an extra staff person to assist with immunization, this individual is not trained to administer vaccines, which creates a bottleneck during immunization sessions. A health facility official noted that some individuals are overburdened with multiple responsibilities. “In each clinic we have two people [for routine immunization]. They are the same people that carryout clinical work in the hospital, and when they leave…we will be closed (H5).” A national level official noted that although the number of recommended vaccines has increased since the country’s immunization program was begun, the human resources to deliver these vaccines has not risen proportionately.

Officials at all levels of government expressed interest in learning more through trainings and on-the-job instruction. Trainings were viewed by some as motivational and a reward for good performance. Most officials claimed that the trainings they had participated in were beneficial and helped to improve their performance. Health facility officials reported recent participation in several trainings, including those for mass immunization campaigns, preparation for introduction of new vaccines (eg, pentavalent vaccine), and maternal, newborn, and child health (MNCH) and integrated community case management (ICCM) programs. One area of training mentioned frequently, especially by LGA officials, but also those at higher levels of government, was data management and computer use.

##### 3. Supplies

Health facility respondents also reported shortages of vaccines and supplies (especially immunization data collection forms). This sometimes leaves officials with no choice but to turn caregivers and children away, at which point women “will be discouraged and…won’t come [back] again” for their child’s vaccinations (H2). LGA officials cited problems with maintaining and powering cold chain equipment, especially with inconsistent electricity from the power grid, insufficient funding for generator fuel, or damaged equipment. An LGA official, for example, reported using ordinary refrigerators that were not part of the program to keep vaccines cool when the cold chain equipment was out of service. A state official mentioned feeling demotivated when LGA officials travel from a far distance to pick up vaccines only to be told that the required vaccines are not available. Officials at LGA, state, and national levels of government cited needing a “little support [with] the modern challenges,” including computers and insufficient internet access for stock management, sending reports, and other communications (S3). An LGA official noted that the recent switch to electronic data entry alleviated delays caused previously due to stockouts of paper data collection forms. Challenges related to infrastructure, namely the limited availability of electricity through the national power grid and the poor road network, were mentioned briefly by several state and national officials.

A national level official noted that financing for immunization is embedded in the budgetary processes of the national government, and because vaccines must be procured months in advance, delays in release of healthcare funding from the federal government can lead to unavailability of vaccines at the state level. State level officials echoed this sentiment as a reason for delays, which they explained are then propagated to lower levels of government.

##### 4. Data

Officials at lower levels of government explained that supervisors visit their facility to correct mistakes and incorrect practices of data collection, summarization, and submission (related to timeliness). The extent to which reported data is analysed at higher levels of government and feedback to LGAs and health facilities was not clear. However, LGA and state officials review immunization coverage and vaccine and logistics data for planning purposes and to address problems. An LGA official gave an example, saying, we have “low coverage in this ward, and…their problem [is] that this clinic didn’t come for vaccines or didn’t do immunization for the month. After I submit the data, [my supervisor] will say I should call” the facility to determine why the missed sessions occurred (L1). Although officials said that they report data on the number of immunizations delivered in their facility every month, this does not necessarily lead to improved availability of vaccines and supplies. An official explained that the recent introduction of the vaccine dashboard, a new system for vaccine stock management, has helped to alleviate this situation. In the “past few months things have improved with this vaccine dashboard…it helps me monitor my stock levels at the LGAs…and call to order any erring LGA leaders as soon as they bring in their reports (S1).” A national official agreed that the dashboard has helped fulfill their responsibilities related to vaccine stock management.

### Axis of Power

#### Incentives and Motivation

Motivation among officials was affected by many of the same areas of accountability emphasized above, but especially the availability of inputs, such as vaccines, infrastructure, equipment, supplies, transportation, and funding. Respondents at all levels of government reported being frustrated and demotivated by their inability to carryout their responsibilities due to these lapses. Several officials stated that simply ensuring the availability of inputs to fulfill their responsibilities would provide sufficient motivation. A state level official specifically mentioned feeling motivated by recent improvements in stock and data management through the vaccine dashboard system. A few officials mentioned “being overloaded” with work as demotivating (N4). Participants tended to discuss motivation in two contexts, how to motivate or incentivise high-performers, and how to ensure a positive working environment.

Suggestions for how motivate high-performers or increase motivation generally included both financial and non-financial strategies. A few LGA and health facility officials suggested raising salaries and increasing clinic and office space, although others at multiple levels of government were content with their salaries. A state official said, “I don’t want incentives to be financial, [just] give me enough airtime to report…and a computer [so] I can [send] feedback to the LGAs.” The same official explained that, outside of mass immunization activities, they have little funding for logistics and office costs (S1).

Officials at the health facility level mentioned individual or health facility recognition for high-performance as motivating. Officials suggested incentives in the form of recognition through “congratulatory messages,” a “certificate to show that I have tried,” or “receiving either verbal [appreciation] or an award related to what you are doing, [so] you know you are doing a good job (S2, H2, L4).” A national level official noted that to properly recognize high-performers, roles and responsibilities need to first be clearly defined, to serve as a benchmark for performance of health workers.

Other suggestions for improved motivation from respondents at all levels of government included training or promotion opportunities for high-performers. A national level official suggested, instead of monetary incentives, focusing on capacity building and “identifying people that have really done well and elevating them to a level [where]…they will push further (N4).”

Supervision also played a role in raising the level of motivation among officials more generally. An official at the LGA level mentioned increased supportive supervision as a potential motivator. LGA and health facility officials were discouraged by negative attitudes of their supervisors, especially the occurrence of shouting when supervisors visit to correct mistakes. An LGA official reported that when supervisors “promise visiting…a health facility, and…don’t come, [this] will not motivate [health workers] (L1).” Two officials, at the LGA and national level, recognized the importance of quality supervision, recognition for good work, inclusion in decision-making, and “personal relationships” on the motivation of their supervisees (N4).

Respondents seemed to highly value, and reported being motivated by, the humanitarian aspect of their work, particularly the ability to provide services that benefit women and children. Attitudes and actions of the community can clearly affect the motivation of health workers, both positively and negatively. A state official said, “my motivation is when I hear or see that the risk for polio is actually dropping, that means we are getting something done (S2).” An LGA official explained, “I love my job, especially [working with] the pregnant women and children. They want someone who is able to help them, so the passion for them is one of the motivating factors (L3).” Turning away mothers and children due to vaccines shortages was mentioned as highly demotivating. The same LGA official reported as demotivating the poor “attitudes of the workers (L3).”

A national official recognized many of the motivating and demotivating factors reported by officials at lower levels of government, saying “I…feel a good working environment [is needed] for [officials] so they won’t scramble for field assignments to earn extra money and training. Other basic needs should be [available], [including] salaries flowing regularly, [and officials should not be] struggling for water, electricity, and adequate security (N3).” This official continued, “generally motivation is very low in government because of [the lack of] these basic needs (N3).” This official added that incentives will only function as incentives when salaries are sufficient and received in a timely manner. Another national level official suggested that they “put [in place] the processes that will clearly define roles and responsibilities, improve on the capacity of people along the line, and recognize good performances (N4).”

#### Penalties and Sanctions

LGA and health facility officials generally reported that they had few problems holding their supervisees accountable for their responsibilities or with other related issues, such as absenteeism. However, when faced with a poor performing supervisee, supervisors described experiencing both positive and negative outcomes after attempting to discuss the problem with the supervisee. One health facility official explained that when they have an underperforming staff person, “I will talk to them and [ask] them to behave, [I have] never had any case where they repeat” the behavior (H2). An LGA level official described the difficulties with addressing poor performance in an environment where the possibility of enforcing penalties is low: “you find something happening and you try to address it, but the person is not ready to comply, and you try to sort it [out] at your level, [but they still] refuse (L4).” A state official explained that the lack of sanctions negatively impacts the process of supervision: “I visited a health facility two or three times, [and suggested] corrections, [but when I] went back, [I] found the same thing on the ground again. I indicated [this] in my report, but no action was taken. It’s so demoralizing (S1).”

No health facility officials interviewed had ever reported anyone to their own supervisors for poor performance or negligence. When LGA and state officials mentioned filing such reports, serious action was typically not taken. A listing of penalties for civil servants does exist, but many said that it is not frequently utilized. Only a few participants could recall any instance where penalties, such as withholding salary or suspension, were enforced. Yet despite how rarely penalties are applied, officials did describe these penalties as effective when enforced. One LGA official recalled the impact of previous cases of suspension and withholding of salary: “when you punish [a supervisee], they come back to their senses…and perform better (L3).”

Officials suggested that the ability to cut salaries, transfer, suspend, or fire employees should be more of a real possibility for penalties to have an impact on performance. It was noted that, for penalties to be effective, enforcement would need to be unhindered by the connections the poor performer has to influential people in the system or community. A national level official also explained that establishment and enforcement of penalties requires certain enabling structures, such as clearly defined roles, responsibilities, standards, and performance targets.

### Axis of Justice

#### Political Leadership, Community Ownership, and Social Equity

LGA and health facility officials most frequently interacted with political or community leaders when problems arose, or when announcing news of a pending an immunization campaign or outreach session to the community. In general, immunization officials felt that support of politicians or community leaders was inconsistent, and very much dependent upon the interest of individual leaders in immunization and PHC. Several remarked that political and community leaders could be more active in supporting the immunization system, especially in educating the community about immunization and encouraging community demand for services. Of note is that any regular effort to engage with community leaders is not without cost. One official explained that funding for the food required to host a meeting with community leaders is frequently unavailable. This official explained that he has to appeal to community leaders on the behalf of the community’s children to secure their attendance.

A state official described a new series of quarterly meetings between immunization officials and LGA chairmen with the purpose of improving coordination and performance in the immunization system. Another similar set of meetings is being held for immunization officials and traditional leaders. This official said that these meetings raise the profile of immunization because the governor is involved. However, another state official stated that the pressure created does not extend beyond the meeting. A national level official also remarked on the importance of this type of effort to “sensitize” governors to the importance of immunization and PHC because they hold the authority for funding allocation and disbursement (N3).

##  Discussion

This qualitative exploratory study examined how different aspects of accountability relate to the performance of the routine immunization system across levels of government in Nigeria. Much of the the discussion with participants centered on the axis of ability, especially inputs and capacity, but also roles, responsibilities, standards, and supervision. The axis of power, which includes incentives and motivation, penalties and sanctions, and decision space and managerial discretion, also featured prominently in the responses. The axis of justice, namely political leadership, community ownership, and social equity, had the least coverage. This may indicate that, according to immunization officials, inputs, capacity, roles, and responsibilities – the fundamental needs that support and enable change in the health system – are more pressing challenges for routine immunization in this region of Nigeria than other areas of accountability that are predominately dynamic or catalytic. Alternatively, this could reflect a common conceptualization of accountability observed in this study, especially at lower levels of government, that accountability concerns accounting for physical vaccine stock, which may have limited the discussion of other themes. An overarching theme evident in the findings of this study, and echoed by a review of similar studies, is the large extent to which the elements of accountability outlined in our framework overlap and reinforce each other.3 In our findings we highlight the division between areas that support and enable change, such as inputs, capacity, roles, and responsibilities, and those that are dynamic or catalytic, such as management practices, decision-making, incentives, and sanctions. In responses from participants, it was often difficult to determine whether the problems discussed were driven primarily by lack of inputs, for example, vaccines, or by the failure of management processes to identify where these vaccines are needed and facilitate their delivery. The value of the accountability framework is to illustrate how these areas interact, either in cases of poor performance, or as the starting point for strategies to improve the functioning of the system. One example is the vaccine stock management “dashboard,” whose early success was cited in several contexts by immunization officials in this study. The dashboard demonstrates how clarifying roles and responsibilities and ensuring the availability of vaccine inputs, within a context of open communication and frequent supervision, can translate into gains in transparency, answerability, and performance. On the surface, officials credited the dashboard with achieving its intended purpose, increasing vaccine availability and reducing stockouts. More fundamentally, however, the tool functioned by alleviating obstacles to accountability, ie, by increasing the visibility and use of data and allowing state managers a quick and effective avenue for supervision of local immunization officials through phone calls, whose purpose was to correct mistakes in vaccine stock balances before new orders are placed and delivered.

Poorly defined roles, responsibilities, and standards were identified here, and in other studies, as an impediment to accountability and high performance within the immunization system and other areas of the health sector in Nigeria.^19,34^ Previous reviews found that the definition of roles and standards can influence the effectiveness of health committees and external accountability on the responsiveness and quality of health services.^35,36^ Undoubtedly, clear roles, responsibilities, and standards are also essential for government officials in positions of technical and managerial leadership.^3,13^

In our study, roles and responsibilities were primarily discussed as elements that support and enable change in the system, the absence of which leads to “confusion and wasted effort.” Overlapping relationships were identified between responsibilities of both officials and institutions and several other accountability areas in the routine immunization system, including inputs (especially funding), supervision, decision space and managerial discretion, and penalties and sanctions. Officials in Niger State raised this point in other discussions, explaining that understaffing and inconsistent funding force supervisors and healthcare workers to assume multiple and shifting roles that create confusion and disrupt regular activities.^37^ Another example of this, offered by a number of respondents in this study, was that supervisors found it difficult to hold their staff accountable and enforce penalties for poor performance without an official listing of roles and responsibilities. Here the operation of a sanction – an enforcement process designed to encourage accountability and discourage negligence – is hindered by the absence of clear and explicit roles and responsibilities, fundamental elements for any PHC system. Nigeria’s Primary Health Care Under One Roof initiative, discussed above, is designed to clarify roles and responsibilities and realign accountability structures through the establishment of State Primary Healthcare Development Agencies.

Inputs and capacity were another major focus of the discussions with participants, and an area critical to accountability and service delivery recognized by previous studies in Nigeria and other countries.^3,19,21,38,39^ Insufficiencies in fundamental inputs that enable accountable operation of the immunization system, such as vaccine supply, data collection forms, and funding, interacted in important ways with the other areas of accountability, for instance, when insufficient funding prevented officials from conducting supervision visits to health facilities. We also found this relationship operating in the opposite direction, evident in instances where officials at lower levels of government lacked the authority to release funding for immunization activities. One important input, the salaries of immunization officials, was not mentioned by respondents, although the timely provision of salaries has been reported as a major challenge for health workers in other regions of Nigeria.^40^

Funding was the most critical of all the inputs mentioned by respondents. In Nigeria, public funding for healthcare, education, water and sanitation, and other services is divided according to a predetermined formula among the three levels of government. Federal statutes mandate funding allocations to designated accounts in each state, and state officials determine subsequent allocations for each LGA based upon criteria such as population size, social development, and other factors.^41^ LGAs are responsible for earmarking and releasing funding for specific purposes, and many LGAs have not established a budget-line for PHC.^30^ Delays in the release of funding can occur at any point in this system, and at the LGA level, availability of funding for PHC is largely reliant upon the highest elected local official, the LGA chairman.^42^ Policies and strategies to improve the regularity of funding have been proposed, including ensuring a budget-line for routine immunization, consolidation of responsibility for access and disbursement of funding through a single state level agency (ie, State Primary Healthcare Development Agency), and state “basket funds.”^30^ Basket funds pool funding from various government, donor, and private sector sources, and serve to ensure adequate, timely, and transparent resources are available to the immunization system.^42^ In another forum, Nigerian immunization officials in Niger State proposed new approaches to improve timely delivery of funding, such as regular electronic banking transfers of funding for immunization and PHC to health facilities, and including an indicator for reporting on funding disbursements in the immunization data reporting system.^37^

Supervision functioned to review system performance, generate motivation, and create a space for increased communication and problem solving. According to respondents, the regularity of supervision visits should be increased, but simply ensuring that supervision visits take place will not likely be sufficient. An immunization official from Niger State remarked in another forum that their inability to solve the problems of the health facilities under their supervision led them to stop visiting these facilities to avoid hearing repeated grievances and having to admit that they could not offer a solution.^37^ Officials in this study declared the need for a supervision checklist, despite the fact that such documents exist for routine immunization supportive supervision visits at both the LGA and state levels in Nigeria.

Supervision is a complex and dynamic process for creating change, and our data, and previous research, suggest that the effectiveness of supervision maybe be particularly reliant upon other factors in the health system, including other areas of accountability.^43,44^ An effective supervision process will require senior actors that prioritize making supervision visits happen; the availability of key inputs such as vaccines, funding, and cold chain equipment; effective downstream processes for reporting and solving problems once they are identified through the supervision process; and the true threat of sanctions for negligence (eg, absenteeism).

Other elements of accountability were also recognized in this study. Decision space and managerial discretion of immunization officials was frequently restricted and arbitrarily defined. This was due in part to the lack of clear roles and responsibilities, and the authority of powerful actors in the system, such as the LGA chairman. Incentives and motivation and penalties and sanctions were identified as useful processes to encourage good performance and discourage negligence, especially in the face of the many challenges encountered by health workers in their daily experiences. Respondents reported on the important influence of local politicians, community leaders, and in some cases, community members, in the management of the immunization system at the local level.^2,7,11,45^

Our study of accountability in the routine system in Nigeria has several limitations. This was a rapid exploratory study of the accountability challenges including immunization officials from only one state and LGA. Participant experiences were not necessarily representative of those in other areas of the country. Participants had varied understandings of accountability, dependent largely on their level of government, making synthesis of the accountability challenges facing the system as a whole more difficult. The study focused primarily on internal accountability, excluding the influence of potentially important interactions between the health system, communities, political leaders, and other stakeholders. Coverage of informal norms was also limited in the discussion on accountability and routine immunization with participants, and should be the topic of future research.

## Ethical issues

The study was reviewed and exempted by Johns Hopkins School of Public Health’s Institutional Review Board, Baltimore, MD, USA and the National Health Research Ethics Committee of Nigeria (NHREC), Abuja, Nigeria.

## Competing interests

Authors declare that they have no competing interests.

## Authors’ contributions

DJE, ASG, CU, CW contributed to the design of the study. DJE and CU carried out the data collection. DJE and ASG conducted the data analysis. DJE, ASG, and CW contributed to the interpretation of the results and drafting the manuscript.

## Authors’ affiliations

^1^Department of International Health, Johns Hopkins Bloomberg School of Public Health, Baltimore, MD, USA. ^2^School of Public Health, University of the Western Cape, Cape Town, South Africa. ^3^i+Consortium, Abuja, Nigeria. ^4^International Vaccine Access Center, Johns Hopkins School of Public Health, Baltimore, MD, USA.

## Key messages

**Implications for policy makers**

- Routine immunization coverage in Nigeria has remained low, and studies have identified a lack of accountability as a barrier to high performance in the immunization system.
- Accountability can be conceptualized as dynamic dimensions framed by three counterbalancing axis: power to spark change; ability to support change; justice to steer change.
- Nigerian health officials highlighted several accountability challenges – especially inconsistent availability of vaccines and funding, unclear roles and responsibilities, poor coordination across levels of government, limited decision space and managerial discretion of lower level officials, and need for increased supervision – which overlap and reinforce each other to prevent high performance of the routine immunization system.
- As an important and knowledgeable stakeholder in ensuring the performance of health systems, views of routine immunization officials should to be prioritized if emerging interventions to improve accountability in routine immunization are to have a positive effect.
- As Nigerian authorities endeavour to improve immunization service delivery, and strengthen the health system more broadly, research should aim to evaluate these programs and policies to provide insight into how to address accountability challenges facing the health sector.

**Implications for public**

Successful delivery of vaccines to health facilities and communities requires coordination among many health officials at the national, state, and local levels that together compose the health system. Immunization coverage in Nigeria is low, and research has attributed this shortcoming to a lack of accountability, in other words, an inability to clearly define roles and responsibilities; provide inputs like vaccines and funding; motivate health officials and enforce penalties when necessary; and include all of the relevant stakeholders, such as community members, political leaders, and government health officials. Through interviews with health officials in Niger State in north central Nigeria, this study identified unclear roles and responsibilities and unavailability of inputs as key obstacles to high performance of the immunization system. Improvement of accountability will play a central role in the efforts of Nigerian authorities to improve immunization coverage, and research should aim to evaluate new programs and policies.
